# If we should remove internal fixation devices for rib fractures?

**DOI:** 10.1186/s13019-023-02330-1

**Published:** 2023-07-04

**Authors:** Yang Li, kaile Jiang, Tiancheng Zhao, Xiang Guo, Kaibin Liu, Yonghong Zhao

**Affiliations:** Department of Thoracic surgery, Shanghai Sixth People’s Hospital, Shanghai, China

**Keywords:** Rib fracture, Removal of internal fixation, Implant-related complication, Postoperative complications

## Abstract

**Background:**

Internal fixation for rib fractures has been widely carried out worldwide, and its surgical efficacy has been recognized. However, there is still controversy about whether implant materials need to be removed. At present, the research on this topic is still lacking at home and abroad. Therefore, in this study, the patients undergoing removal of internal fixation for rib fractures in our department within one year were followed up, to statistically analyze implant-related complications, postoperative complications and postoperative remission rate.

**Methods:**

A retrospective analysis was conducted on 143 patients undergoing removal of internal fixation for rib fractures from 2020 to 2021 in our center. The implant-related complications, postoperative complications and postoperative remission rate of patients with internal fixation were analyzed.

**Results:**

In this study, a total of 143 patients underwent removal of internal fixation, among which 73 suffered from preoperative implant-related complications (foreign-body sensation, pain, wound numbness, sense of tightness, screw slippage, chest tightness, implant rejection), and 70 had no post operative discomfort but asked for removal of internal fixation. The average interval between rib fixation and removal was 17 ± 9.00 (months), and the average number of removed materials was 5.29 ± 2.42. Postoperative complications included wound infection (n = 1) and pulmonary embolism (n = 1). of the 73 patients with preoperative implant-related complications, the mean postoperative remission rate was 82%. Among the 70 patients without preoperative discomfort, the proportion of discomforts after removal was 10%. No perioperative death occurred.

**Conclusion:**

For patients with internal fixation for rib fractures, removal of internal fixation can be considered in the case of implant-related complications after surgery. The corresponding symptoms can be relieved after removal. The removal presents low complication rate, and high safety and reliability. For patients without obvious symptoms, it is safe to retain the internal fixation in the body. For the asymptomatic patients who ask for removal of internal fixation, the possible risk of complications should be fully informed before removal.

## Background

With the surgical concept of internal fixation for rib fractures becoming increasingly popular, internal fixation for multiple rib fractures has been carried out more and more widely [1, 2]. Along with the increase in surgical numbers, the following problem is whether the internal fixation devices of the ribs need to be removed after a certain period of time. In the technical documents provided by manufacturers, it is suggested that the internal fixation devices should be removed at an appropriate time, and the timing and indications should be determined by clinicians, but no corresponding documentation has been provided. In the existing literature, there is almost no discussion about the removal of internal fixation devices for rib fractures. Reviewing the orthopedic literature on the removal of internal fixation devices, there are considerable differences of opinion on whether internal fixation devices need to be removed in asymptomatic patients, and high-level evidence-based medical evidence is lacking to determine whether it is necessary to remove internal fixation devices for fractures [3, 4].

In actual clinical practice, a considerable number of orthopedic internal fixation devices have been removed, but the surgical indications are still quite vague. In a Finnish study, nearly all (81%) implants inserted for fracture fixation were eventually removed [5]. In total, implant removal contributed to almost 30% of all planned orthopaedic operations [6]. The reason for this perception and behaviour mismatch remains unclear particularly considering the associated high complication rate of 20% with up to 14% infection rate, 2% nerve injury and re-fracture in 0.5% [7].

We statistically analyzed the patients undergoing removal of internal fixation devices for rib fractures in the Department of Thoracic Surgery of Shanghai Sixth People’s Hospital in the past year, in the expectation for summarizing and discussing the reasons, risks and benefits of removal of internal fixation devices for rib fractures.

Methods.

The data of patients undergoing removal of internal fixation for rib fractures from 2020 to 2021 in our department were collected for a retrospective analysis. All patients underwent internal fixation with Ni-TI claw plates and Synthes rib fixation plates.The data were sorted out with the trauma database of our department. The basic clinical characteristics of the patients were analyzed. The collected data included the reasons for removal of internal fixation, preoperative symptoms, postoperative symptom remission rate and postoperative complications. The patients were followed up for symptom improvement for 3 months. All the data were analyzed.

## Results

During 2020–2021, 143 patients undergoing removal of internal fixation devices for rib fractures in our department. The basic clinical information is seen in Table 1. There were 75 males and 68 females, with an average age of 56 ± 2.32 years. Between internal fixation and removal was 17 ± 9.00 months. In the same period, a total of 554 patients underwent internal fixation for rib fractures, and the proportion of patients undergoing removal of internal fixation was about 25.81% of all patients undergoing surgery in the same period. Among them, 70 (48.95%) patients had no symptoms but asked for removal of internal fixation, and 73 (51.05%) patients suffered from implant-related complications and required removal of internal fixation.

**Table 1:** Basic data

	Number
Number of cases	143
Average age	57 ± 4.25(y)
Gender	
Male	75
Female	68
Mean time between operations	17 ± 9.00 (m)
Average number of implants removed	5.29 ± 2.42
Reasons for surgery	
Asymptomatic patients	70
Foreign-body sensation	38
Pain	21
Numbness	10
Tightness	1
Screw slippage	1
Chest tightness	1
Implants rejection	1
Complications	
Wound infection	1
Pulmonary infection	0
Pulmonary embolism	1
Death	0

A statistics was also performed on the reasons for surgical removal of internal fixation, as shown in Table 2. Among all the patients with symptoms undergoing removal, the most common symptoms were pain and foreign-body sensation in the chest (n = 59), accounting for 80.82%. Other discomforts included sense of numbness and tightness (n = 11), accounting for 15.06%. Additionally, there was 1 patient requiring removal of internal fixation due to poor wound healing caused by screw slippage, chest tightness and implants, respectively. The common complications during and after removal included re-fracture and wound infection. There were 2 cases of wound infection and 3 cases of secondary fracture caused by intraoperative bone injury. In all the patients, one developed pulmonary embolism after removal, which was improved after active treatment. No death occurred among all the included patients.

**Table 2:** Postoperative status of patients with preoperative symptoms

Reasons for surgery	Number	Postoperative improvements	
Foreign-body sensation	38	36	94.74%
Pain	21	13	61.90%
Numbness	10	7	70.00%
Tightness	1	1	100.00%
Screw slippage	1	1	100.00%
Chest tightness	1	1	100.00%
Implants rejection	1	1	100.00%
Total	73	60	82.19%

Moreover, a 3-month follow-up was carried out on the improvement in patients’ symptoms after removal. In all the symptomatic patients undergoing removal, the overall improvement was 82.19%(Table 2). Among the 70 asymptomatic patients, 2 had chest wall pain after removal, and 3 presented a sense of tightness (Table 3).

**Table 3:** Postoperative status of patients without preoperative symptoms

	Number
No symptoms after surgery	65
Symptoms after surgery	
Pain	2
Numbness	0
Tightness	3
Chest tightness	0
Postoperative complications	0
Total	70

## Discussion

Surgical internal fixation has been generally accepted to be the most effective treatment for multiple rib fractures or flail chest. Our previous study has also confirmed that surgical internal fixation can rapidly relieve the respiratory impairment and pain caused by rib fractures [8]. However, doctors and patients in different countries have distinct perceptions about whether the internal fixation devices need to be removed. As the surgical internal fixation for rib fractures has not been carried out for a long time, we lack reference data to support any theory.

According to our study, it was found that the patients undergoing removal of internal fixation could be divided into two groups. One group without any discomfort concerning about the implants retained in the body due to social and cultural factors, and still hoped to remove the implants. In our study, most of these patients recovered well after removal of internal fixation without obvious postoperative complications, but 2 presented chest wall pain and 3 had a sense of tightness around the wound after removal. The possible reason lies in that the bone may be damaged to a certain extent during the operation, resulting in postoperative chest pain [9]. The appearance of the sense of tightness around the wound is firstly considered to be resulted from local contracture caused by scar healing. The probability of secondary trauma will be increased in removal of internal fixation to a certain extent [10].

For the other group with discomforts, internal fixation for rib fractures would still bring some adverse effects to partial patients, including pain, foreign-body sensation and discomforts. Although the proportions were not high, they would still affect the quality of life of the patients. Based on our data, the majority of the symptomatic patients had obvious remission of their discomforts after removal of the internal fixation. Among them, some symptoms of the patients with a preoperative sense of numbness and tightness were relieved after implant removal. Therefore, we consider that this sense of numbness and tightness is not only caused by the nerve terminal injury and scar healing caused by surgery, but also related to the implantation of internal fixation materials [11]. Other similar chest tightness, foreign-body sensation and poor wound healing were significantly improved after removal of the internal fixation. Consequently, for these patients, we believe that the removal of internal fixation is still necessary and valuable.

According to our data, the incidence of removal-related complications was about 4.2%. The common complications was secondary fracture caused by wound infection and bone injury. In previous studies, it was proposed that most removal of internal fixation were performed by junior physicians, so more removal-related complications might occur. In general, we believe that the removal of internal is safe with low incidence of complications, which can be treated.

## Conclusion

In conclusion, for patients with internal fixation for rib fractures, removal of internal fixation can be considered in the case of chest discomforts after surgery, including pain, sense of tightness and foreign-body sensation. The corresponding symptoms can be relieved after removal. The removal presents low complication rate, and high safety and reliability. For patients without obvious symptoms, it can be considered to retain the internal fixation in the body. For the asymptomatic patients who ask for removal of internal fixation, the possible risk of complications should be fully informed before removal.

## Data Availability

Data and materials are available by e-mail.
